# Complete Genome Sequence of *Annamia dubia*, filamentous colony-making Chroococcales with the analysis of FraC gene influencing filament integrity

**DOI:** 10.7150/jgen.87678

**Published:** 2024-01-01

**Authors:** Akihiro Tuji, Eri Ogiso-Tanaka, Haruyo Yamaguchi

**Affiliations:** 1 Department of Botany, National Museum of Nature and Science, Amakubo 4-1-1, Tsukuba, Ibaraki, 305-0005, Japan.; 2 Center for Molecular Biodiversity Research, National Museum of Nature and Science, Amakubo 4-1-1, Tsukuba, Ibaraki, 305-0005, Japan.; 3 Biodiversity Division, National Institute for Environmental Studies, 16-2 Onogawa, Tsukuba, Ibaraki 305-8506, Japan.

## Abstract

The complete genome of *Annamia dubia* was sequenced. The genome size is 4.02 Mbp, including 3886286 bp circular chromosome and four circular plasmids (31516, 42453, 38085 and 24903 bp). It included 3718 protein-coding sequences, 45 tRNA genes, three sets of rRNA genes, a microcystin biosynthesis gene cluster and six CRISPR (clustered regularly interspaced short palindromic repeat). *Annamia* is the only one genus in the Chroococcales that makes filamentous colonies. FraC and FraG were identified in the genome. These genes are required for the integrity of cell junctions and influencing filament integrity and are thought to be related to colony formation. These genes are first reported from Chroococcales, and may play a significant role in the colony formation of this species.

In the phylogenetic tree of the FraC gene, *A. dubia* was located in the basal position of Oscillatoriales. The GC ratio of FraC gene of *A. dubia* is very low from the genome and the FraC gene of Microcoleaceae. The presence of these genes in the basal region and the low GC ratio suggests that the FraC gene in this species was introduced by horizontal gene transfer. Since the filamentous colony is a fundamental and important taxonomic feature for the classification of cyanobacteria, the possibility of horizontal transmission of genes involved in filamentous cyanobacterial colonies is an important discovery for the classification of cyanobacteria.

## Introduction

The genus *Annamia* was described with the type species *A. toxica*. This genus was assigned to the family Borziaceae within the order Oscillatoriales 1, because it has radially arranged thylakoids and forms filamentous colonies like the genus *Pseudanabaena*. Tuji et al. 2 described the second member of this genus as *A. dubia* from Lake Kasumigaura Japan. In the phylogenetic tree using 16S rRNA and ITS region, the genus *Annamia* matched the clade that had been assigned to 'Cyanobacteriaceae' 3, including the genera *Cyanobacterium*, *Geminobacterium* and *Geminocystis*. Due to International Code of Nomenclature for algae, fungi, and plants (ICN) rule issues, 'Cyanobacteriaceae' was renamed Geminocystaceae 1 within the order Chroococcales 2. The order Chroococcales has eight families 4 which are characterized by coccoid forms or pseudo-filaments with sheaths 3 5 and irregular thylakoid arrangement 3. The genus *Annamia* is only one exception to the order Chroococcales in forming true filamentous colonies. No cultured strains of *A. toxica* remain 1, and a cultured strain of* A. dubia* (NIES-4383) is the only cultivated strain of this genus. This genus is also an exception in the Chroococcus family, which contains Microcystin. In this study, we performed a whole genome analysis of *A. dubia* (NIES-4383) with these unique characteristics and studied its phylogeny with closely related families.

## Materials and Methods

A culture strain of *A. dubia* (NIES-4383) 2 was used in this study. DNA extraction from a 200 mL culture of *A. dubia* (NIES-4383) was performed using the lysis solution and magnetic beads separation 6. DNA sequencing was performed using a MinION sequencer (Oxford Nanopore Technologies, Oxford, UK) and Illumina MiSeq (San Diego, CA, USA). For Illumina MiSeq sequencing, DNA was fragmented using the Covaris M220 Ultrasonicator (Woburn, MA, USA) to obtain 550-bp reads. The DNA library was prepared using the NEBNext Ultra DNA Library Prep Kit for Illumina (New England Biolabs, Ipswich, MA, USA) following the manufacturer's protocol. Sequencing was performed using the 600-cycle MiSeq Reagent Kit v.3. In total, 5,926,737 paired-end reads (1.75 Gbp in total) were obtained. The generated reads were trimmed for adapters and low-quality reads using Trimmomatic v.0.36 7. For MinION sequencing, a DNA library was prepared using the Ligation sequencing Kit (SQK-LSK109) following standard protocols provided by Oxford Nanopore Technologies (ONT). The MinION MK1C sequencer and flow cell (R9.4.1) were used for sequencing and the generated raw data were basecalled in High-accurate mode using Guppy v3.4.4 (ONT). In total, 159837 reads (960 Mbp) were obtained. The reads were filtered using Filtlong v0.2.0 (minimum length 1000 bp and keep percent 95). Reads were then assembled using the Trycycler pipeline v.0.4.1 7 First, subsampling was performed to generate 12 subset fastq files using the default method provided by Trycycler. Three different assemblers were used to generate 12 draft assemblies by using flye v2.82-b1689 8, miniasm v0.3-r179, minipolish v0.1.3 9 and raven v1.3.1 10. Then, the contigs from all assembled into groups of identical copies and removed incorrectly assembled contigs. The next reconcile step, contigs with too much difference in length within each cluster were removed. After the next sequence alignment and consensus step, the consensus sequences were polished with MiSeq reads using pilon v1.23 11. Four rounds of polishing (BWA-mem aligned short read) were performed. The contaminant-derived contigs were determined by BLAST and removed. The genome was annotated using DFAST 12. A chromosome map of this strain was drawn using DNAPlotter 13. Phylogenetic and molecular evolutionary investigations of the FraC gene sequence, annotated utilizing the DFAST approach, and correlated sequences procured through a BLAST search, were performed employing the MEGA 7 computational software 14. The alignments were checked manually. A maximum likelihood tree was calculated using MEGA software. A tree using 500 bootstrap replicates was generated.

## Results and Discussion

**Figure 1:**
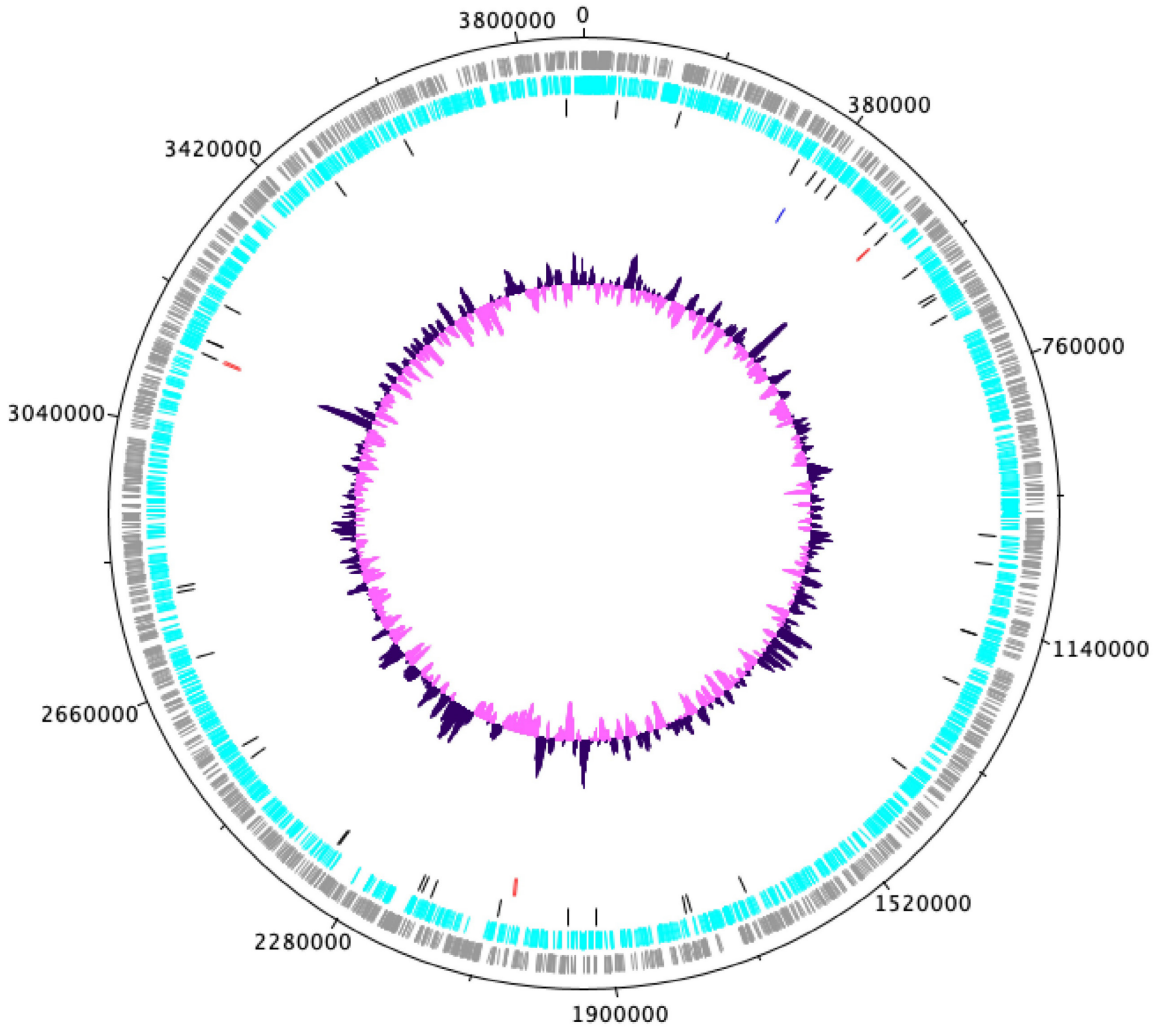
Complete chromosome map of *Annamia dubia* NIES-4383. The chromosome map comprises five concentric circles. The gray and light-blue circles show the positions of protein-coding genes on the plus and minus strands, respectively. Black bars on the third circle, red bars on the fourth circle, blue bar on the fifth circle, and purple/pink circle show tRNA, rRNA genes, Fra genes, and guanine-cytosine content.

Genomic characteristics of *A. dubia* (NIES-4383) are summarized in Table 1. We obtained a genome consisting of a 3886286 bp circular chromosome (Fig. 1). It included 3475 protein-coding sequences, 45 tRNA genes, six sets of rRNA genes and eight CRISPR (clustered regularly interspaced short palindromic repeat). The G+C content was 33.28%. Four circular plastid genomes of 42453, 31516, 38085 and 24903 bp were also obtained. Obtained other two circulars are regarded as contamination, because the result of Blast search and its depth, and these are omitted from further analysis. Nanopore MinION and illumina MiSeq read coverage were 239-fold and 434-fold, respectively.

**Figure 2:**
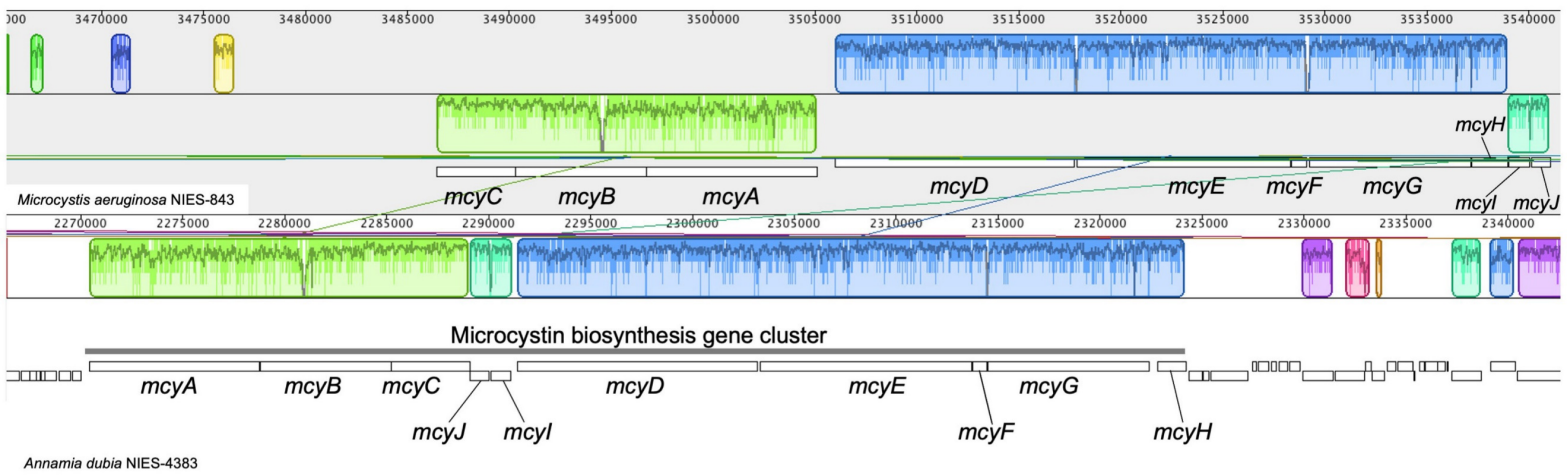
Comparison of the microcystin biosynthesis gene cluster between *Microcystis aeruginosa* NIES-843 and *Annamia dubia* NIES-4383. The gene order of the cluster is very similar except for the reversed order of *mcyA* to *mcyC*. The figure was drawn using Mauve software (http://darlinglab.org/mauve/mauve.html).

*A. dubia* produces a hepatotoxin, microcystin 15. The toxin is generated through a multifunctional enzyme complex, which includes both peptide synthetase and polyketide synthase modules encoded by the microcystin biosynthesis gene cluster. The microcystin biosynthesis gene cluster is widely distributed in the genus *Microcystis* 16. In *Microcystis aeruginosa,* the genes comprising the microcystin biosynthesis cluster are well conserved. In *M. aeruginosa* NIES-843, the genes that compose the cluster are arranged in the order *mcyC*, *mcyB*, *mcyA*, *mcyD*, *mcyE*, *mcyF*, *mcyG*, *mcyH*, *mcyI* and *mcyJ* 17, but in *A. dubia*, they are arranged in the order *mcyA*, *mcyB*, *mcyC*, *mcyJ*, *mcyI*, *mcyD*, *mcyE*, *mcyF*, *mcyG* and *mcyH* (Fig. 2). Comparison of each homologous gene in the cluster using blastp showed that the highest amino acid sequence homology between *A. dubia* and *M. aeruginosa* genes was 88%, and the lowest was 72%. The genes are arraigned in tandem and have the highest amino acid sequence homology suggest the microcystin biosynthesis gene cluster would be derived from *Microcystis* by horizontal gene transfer.

**Figure 3:**
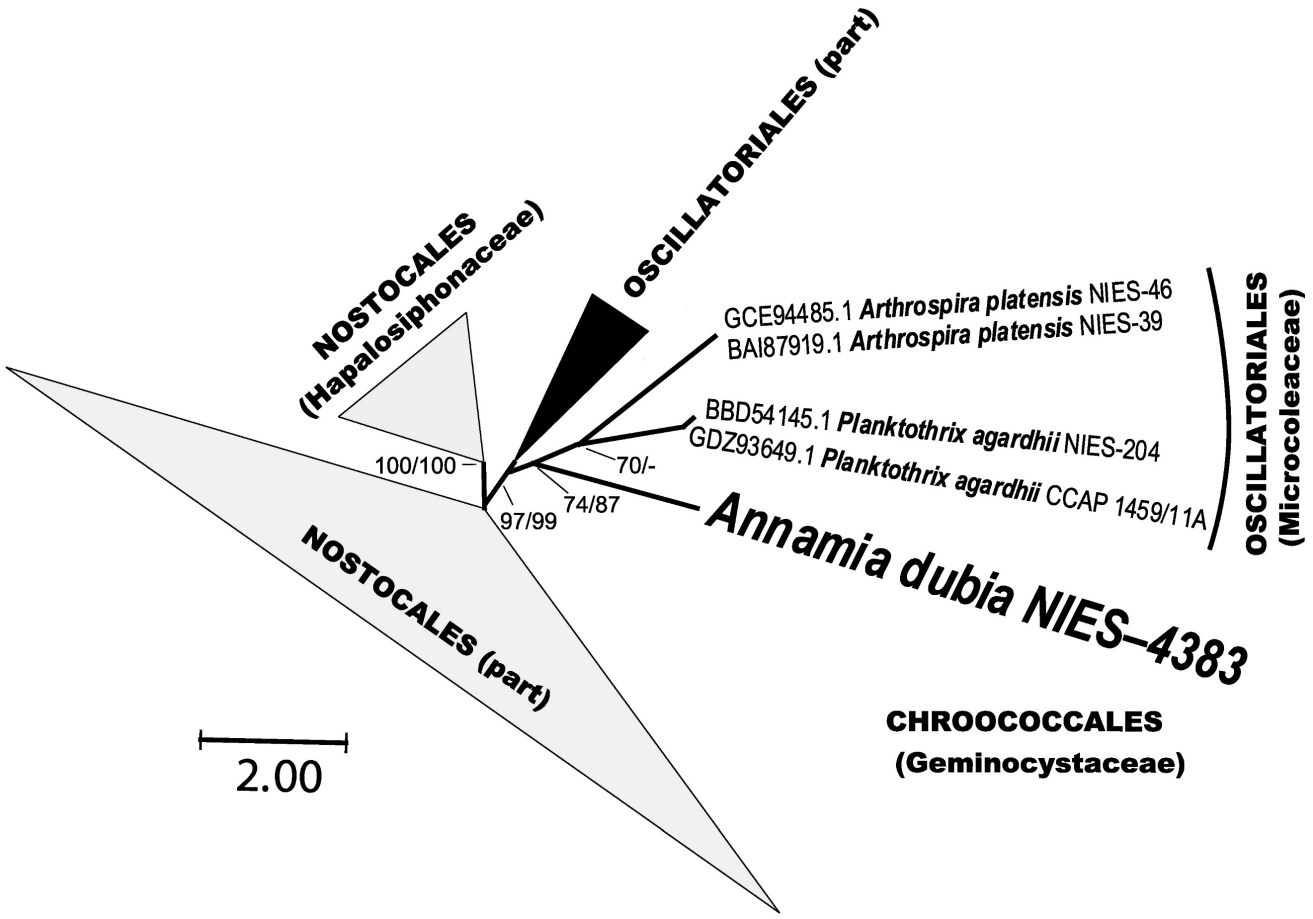
Maximum likelihood phylogenetic tree based on* Annamia dubia* and related taxa of the FraC gene showing the relationship with related orders. Bootstrap support (BS) with NJ and ML methods are indicated at the nodes. A - indicates less than 0.70 support.

FraC and FraG were identified in the genome. These genes are required for the integrity of cell junctions 15 and influencing filament integrity and thought to be the relation with colony formation 18, 19. Consequently, these genes may play a significant role in the colony formation of this species. Owing to the scarcity of information regarding the occurrence of the FraG gene, only the FraC gene was scrutinized in this investigation. The phylogenetic tree of the FraC gene obtained in this study is shown in Fig. 3. This gene was found in Nostocales and Oscilatoriales which make filamentous colonies. *A. dubia* (NIES-4383) is the first species for the FraC gene in Chroococcales.* A. dubia* is exist basal point between Nostocales and Oscillatoriales. The GC ratio of the FraC gene is 0.20 and one of the lowest GC ratio genes in this strain. The GC ratio of genomes for Microcoleaceae are from 44.2 to 44.6, and GC ratio of FraC gene for Microcoleaceae are 36. The GC ratio of FraC gene of *A. dubia* is very low from the genome and the FraC gene of Microcoleaceae. The presence of these genes in the basal region and the low GC ratio suggests that the FraC gene in this species was introduced by horizontal gene transfer. The formation of filamentous colony is a fundamental and important taxonomic feature for the classification of cyanobacteria. The possibility of horizontal transmission of genes involved in filamentous cyanobacterial colonies is an important discovery for the classification of cyanobacteria.

## Figures and Tables

**Table 1:** General characteristics of *Annamia dubia* NIES-4383

Features	*Annamina dubia* NIES-4383				
Accession	AP025630	AP025631	AP025632	AP025633	AP025634
Type	Chromosome	Plasmid	Plasmid	Plasmid	Plasmid
genome size (bp)	3,886,286	31516	42453	38085	24903
GC content (%)	33.28%	32.58%	31.69%	33.22%	33.61%
Number of coding sequence	3475	31	45	35	25
rRNA operon	3	0	0	0	0
tRNA genes	45	0	0	0	0
CRISPRs	8	0	0	0	0
